# Supplementary material for: Diagnostic Performance of Various Ultrasound Risk Stratification Systems for Benign and Malignant Thyroid Nodules: A Meta-Analysis
Source: Cancers (Basel). 2023 Jan 9;15(2):424. doi: 10.3390/cancers15020424 (PMC9857194; doi:10.3390/cancers15020424)
Supplement: Supplementary file 1 [file cancers-15-00424-s001.zip › cancers-2063291-supplementary.pdf]

Supplementary Table S1. Search terms by database

Database	Search	Search terms
PubMed	#1	"Thyroid Neoplasms"[Mesh]
	#2	"Thyroid Neoplasms"[TW] OR "Neoplasm, Thyroid"[TW] OR "Thyroid Neoplasm"[TW] OR "Neoplasms, Thyroid"[TW] OR "Thyroid Carcinoma"[TW] OR "Carcinoma, Thyroid"[TW] OR "Carcinomas, Thyroid"[TW] OR "Thyroid Carcinomas"[TW] OR "Cancer of Thyroid"[TW] OR "Thyroid Cancers"[TW] OR "Thyroid Cancer"[TW] OR "Cancer, Thyroid"[TW] OR "Cancers, Thyroid"[TW] OR "Cancer of the Thyroid"[TW] OR "Thyroid Adenoma"[TW] OR "Adenoma, Thyroid"[TW] OR "Adenomas, Thyroid"[TW] OR "Thyroid Adenomas"[TW] OR "thyroid tumor"[TW] OR "neoplasm of thyroid gland"[TW] OR "neoplastic thyroid"[TW] OR "neoplastic thyroid gland"[TW] OR "thyroid gland neoplasia"[TW] OR "thyroid gland neoplasm"[TW] OR "thyroid gland tumor"[TW] OR "thyroid gland tumour"[TW] OR "thyroid neoplasia"[TW] OR "thyroid tumorigenesis"[TW] OR "thyroid tumour"[TW] OR "thyroidal tumor"[TW] OR "thyroidal tumour"[TW] OR "tumor of thyroid"[TW] OR "tumor of thyroid gland"[TW] OR "tumor, thyroid gland"[TW] OR "tumour of thyroid"[TW] OR "tumour of thyroid gland"[TW] OR "tumour, thyroid gland"[TW]
	#3	"Thyroid Nodule"[Mesh]
	#4	"Thyroid Nodule"[TW] OR "Nodule, Thyroid"[TW] OR "Nodules, Thyroid"[TW] OR "Thyroid Nodules"[TW] OR "colloid nodule (thyroid)"[TW] OR "colloidal nodule (thyroid)"[TW] OR "nodule of the thyroid gland"[TW] OR "nodule of thyroid gland"[TW] OR "nodule, thyroid gland"[TW] OR "thyroid colloid nodule"[TW] OR "thyroid colloidal nodule"[TW] OR "thyroid gland nodule"[TW] OR "thyroid solitary nodule"[TW] OR "thyroidal gland nodule"[TW] OR "thyroidal nodule"[TW] OR "thyroideal nodule"[TW]
	#5	"thyroid mass"[TW]
	#6	#1 OR #2 OR #3 OR #4 OR #5
	#7	"Ultrasonography"[Mesh]
	#8	"ultrasound"[TW] OR "Ultrasonography"[TW] OR "Diagnostic Ultrasound"[TW] OR "Diagnostic Ultrasounds"[TW] OR "Ultrasound, Diagnostic"[TW] OR "Ultrasounds, Diagnostic"[TW] OR "Ultrasound Imaging"[TW] OR "Imaging, Ultrasound"[TW] OR "Imagings, Ultrasound"[TW] OR "Echotomography"[TW] OR "Ultrasonic Imaging"[TW] OR "Imaging, Ultrasonic"[TW] OR "Sonography, Medical"[TW] OR "Medical Sonography"[TW] OR "Ultrasonographic Imaging"[TW] OR "Imaging, Ultrasonographic"[TW] OR "Imagings, Ultrasonographic"[TW] OR "Ultrasonographic Imagings"[TW] OR "Echography"[TW] OR "Diagnosis, Ultrasonic"[TW] OR "Diagnoses, Ultrasonic"[TW] OR "Ultrasonic Diagnoses"[TW] OR "Ultrasonic Diagnosis"[TW] OR "Echotomography, Computer"[TW] OR "Computer Echotomography"[TW] OR "Tomography, Ultrasonic"[TW] OR "Ultrasonic Tomography"[TW] OR "diagnostic ultrasonic examination"[TW] OR "diagnostic ultrasonic imaging"[TW] OR "diagnostic ultrasonic method"[TW] OR "doptone"[TW] OR "duplex echography"[TW] OR "echogram"[TW] OR "echographic evaluation"[TW] OR "echoscopy"[TW] OR "echosound"[TW] OR "high resolution echography"[TW] OR "scanning, ultrasonic"[TW] OR "sonogram"[TW] OR "sonographic examination"[TW] OR "sonographic screening"[TW] OR "sonography"[TW] OR "ultrasonic detection"[TW] OR "ultrasonic echo"[TW] OR "ultrasonic examination"[TW] OR "ultrasonic scanning"[TW] OR "ultrasonic scintillation"[TW] OR "ultrasonogram"[TW] OR "ultrasonographic examination"[TW] OR "ultrasonographic screening"[TW] OR "ultrasound diagnosis"[TW] OR "ultrasound scanning"[TW]
	#9	"Biopsy"[Mesh]
	#10	"Biopsy"[TW] OR "Biopsies"[TW] OR "bioptic diagnosis"[TW] OR "bioptical diagnosis"[TW] OR "rebiopsy"[TW]
	#11	"Biopsy, Fine-Needle"[Mesh]

	#12	"Biopsy, Fine-Needle"[TW] OR "Biopsies, Fine-Needle"[TW] OR "Biopsy, Fine Needle"[TW] OR "Fine-Needle Biopsies"[TW] OR "Fine-Needle Biopsy"[TW] OR "Fine Needle Biopsy"[TW] OR "Biopsies, Fine Needle"[TW] OR "Fine Needle Biopsies"[TW] OR "Needle Biopsies, Fine"[TW] OR "Needle Biopsy, Fine"[TW] OR "Aspiration Biopsy, Fine-Needle"[TW] OR "Aspiration Biopsies, Fine-Needle"[TW] OR "Aspiration Biopsy, Fine Needle"[TW] OR "Biopsies, Fine-Needle Aspiration"[TW] OR "Biopsy, Fine-Needle Aspiration"[TW] OR "Fine-Needle Aspiration Biopsies"[TW] OR "Fine-Needle Aspiration Biopsy"[TW] OR "Fine-Needle Aspiration"[TW] OR "Aspiration, Fine-Needle"[TW] OR "Aspirations, Fine-Needle"[TW] OR "Fine Needle Aspiration"[TW] OR "Fine-Needle Aspirations"[TW] OR "fine needle aspiration biopsy"[TW] OR "fine core needle biopsy"[TW] OR "fine needle aspiration cytology"[TW] OR "fine needle aspirations"[TW] OR "FNA (fine needle aspiration)"[TW] OR "FNAB (fine needle aspiration biopsy)"[TW] OR "FNAC (fine needle aspiration cytology)"[TW] OR "needle aspiration"[TW]
	#13	#7 OR #8 OR #9 OR #10 OR #11 OR #12
	#14	"Risk Assessment"[Mesh]
	#15	"Risk Assessment"[TW] OR "Risk Assessments"[TW] OR "Health Risk Assessment"[TW] OR "Assessment, Health Risk"[TW] OR "Health Risk Assessments"[TW] OR "Risk Assessment, Health"[TW] OR "Assessment, Risk"[TW] OR "Risks and Benefits"[TW] OR "Benefits and Risks"[TW] OR "Risk Analysis"[TW] OR "Analysis, Risk"[TW] OR "Risk Analyses"[TW] OR "Benefit-Risk Assessment"[TW] OR "Assessment, Benefit-Risk"[TW] OR "Benefit Risk Assessment"[TW] OR "Benefit-Risk Assessments"[TW] OR "Risk-Benefit Assessment"[TW] OR "Assessment, Risk-Benefit"[TW] OR "Risk Benefit Assessment"[TW] OR "Risk-Benefit Assessments"[TW] OR "risk stratification"[TW] OR "assessment, safety"[TW] OR "risk adjustment"[TW] OR "risk evaluation"[TW] OR "safety assessment"[TW]
	#16	"Korean Thyroid Association"[TW] OR "Korean Society of Thyroid Radiology"[TW] OR "American Thyroid Association"[TW] OR "American College of Radiology"[TW] OR "European Thyroid Association"[TW] OR "American Association of Clinical Endocrinologists"[TW] OR "Society of Radiologists"[TW] OR "KTA"[TW] OR "KSThR"[TW] OR "ATA"[TW] OR "ACR"[TW] OR "ETA"[TW] OR "SRU"[TW] OR "thyroid imaging reporting and data system"[TW] OR "TIRADS"[TW] OR "TI-RADS"[TW] OR "Kwak-TIRADS"[TW] OR "K-TIRADS"[TW] OR "ACR-TIRADS"[TW] OR "EU-TIRADS"[TW]
	#17	#14 OR #15 OR #16
	#18	#13 AND #17
	#19	#6 AND #18
	#20	#19 NOT ("Animals"[Mesh] NOT ("Animals"[Mesh] AND "Humans"[Mesh]))

Database	Search	Search terms
EMBASE	#1	"thyroid tumor"/exp
	#2	"Thyroid Neoplasms":ti,ab,kw,de OR "Neoplasm, Thyroid":ti,ab,kw,de OR "Thyroid Neoplasm":ti,ab,kw,de OR "Neoplasms, Thyroid":ti,ab,kw,de OR "Thyroid Carcinoma":ti,ab,kw,de OR "Carcinoma, Thyroid":ti,ab,kw,de OR "Carcinomas, Thyroid":ti,ab,kw,de OR "Thyroid Carcinomas":ti,ab,kw,de OR "Cancer of Thyroid":ti,ab,kw,de OR "Thyroid Cancers":ti,ab,kw,de OR "Thyroid Cancer":ti,ab,kw,de OR "Cancer, Thyroid":ti,ab,kw,de OR "Cancers, Thyroid":ti,ab,kw,de OR "Cancer of the Thyroid":ti,ab,kw,de OR "Thyroid Adenoma":ti,ab,kw,de OR "Adenoma, Thyroid":ti,ab,kw,de OR "Adenomas, Thyroid":ti,ab,kw,de OR "Thyroid Adenomas":ti,ab,kw,de OR "thyroid tumor":ti,ab,kw,de OR "neoplasm of thyroid gland":ti,ab,kw,de OR "neoplastic thyroid":ti,ab,kw,de OR "neoplastic thyroid gland":ti,ab,kw,de OR "thyroid gland neoplasia":ti,ab,kw,de OR "thyroid gland neoplasm":ti,ab,kw,de OR "thyroid gland tumor":ti,ab,kw,de OR "thyroid gland tumour":ti,ab,kw,de OR "thyroid neoplasia":ti,ab,kw,de OR "thyroid tumorigenesis":ti,ab,kw,de OR "thyroid tumour":ti,ab,kw,de OR "thyroidal tumor":ti,ab,kw,de OR "thyroidal tumour":ti,ab,kw,de OR "tumor of thyroid":ti,ab,kw,de OR "tumor of thyroid gland":ti,ab,kw,de OR "tumor, thyroid gland":ti,ab,kw,de OR "tumour of thyroid":ti,ab,kw,de OR "tumour of thyroid gland":ti,ab,kw,de OR "tumour, thyroid gland":ti,ab,kw,de
	#3	"thyroid nodule"/exp

	#4	"Thyroid Nodule":ti,ab,kw,de OR "Nodule, Thyroid":ti,ab,kw,de OR "Nodules, Thyroid":ti,ab,kw,de OR "Thyroid Nodules":ti,ab,kw,de OR "colloid nodule (thyroid)":ti,ab,kw,de OR "colloidal nodule (thyroid)":ti,ab,kw,de OR "nodule of the thyroid gland":ti,ab,kw,de OR "nodule of thyroid gland":ti,ab,kw,de OR "nodule, thyroid gland":ti,ab,kw,de OR "thyroid colloid nodule":ti,ab,kw,de OR "thyroid colloidal nodule":ti,ab,kw,de OR "thyroid gland nodule":ti,ab,kw,de OR "thyroid solitary nodule":ti,ab,kw,de OR "thyroidal gland nodule":ti,ab,kw,de OR "thyroidal nodule":ti,ab,kw,de OR "thyroideal nodule":ti,ab,kw,de
	#5	"thyroid mass":ti,ab,kw,de
	#6	#1 OR #2 OR #3 OR #4 OR #5
	#7	"echography"/exp
	#8	"ultrasound":ti,ab,kw,de OR "Ultrasonography":ti,ab,kw,de OR "Diagnostic Ultrasound":ti,ab,kw,de OR "Diagnostic Ultrasounds":ti,ab,kw,de OR "Ultrasound, Diagnostic":ti,ab,kw,de OR "Ultrasounds, Diagnostic":ti,ab,kw,de OR "Ultrasound Imaging":ti,ab,kw,de OR "Imaging, Ultrasound":ti,ab,kw,de OR "Imagings, Ultrasound":ti,ab,kw,de OR "Echotomography":ti,ab,kw,de OR "Ultrasonic Imaging":ti,ab,kw,de OR "Imaging, Ultrasonic":ti,ab,kw,de OR "Sonography, Medical":ti,ab,kw,de OR "Medical Sonography":ti,ab,kw,de OR "Ultrasonographic Imaging":ti,ab,kw,de OR "Imaging, Ultrasonographic":ti,ab,kw,de OR "Imagings, Ultrasonographic":ti,ab,kw,de OR "Ultrasonographic Imagings":ti,ab,kw,de OR "Echography":ti,ab,kw,de OR "Diagnosis, Ultrasonic":ti,ab,kw,de OR "Diagnoses, Ultrasonic":ti,ab,kw,de OR "Ultrasonic Diagnoses":ti,ab,kw,de OR "Ultrasonic Diagnosis":ti,ab,kw,de OR "Echotomography, Computer":ti,ab,kw,de OR "Computer Echotomography":ti,ab,kw,de OR "Tomography, Ultrasonic":ti,ab,kw,de OR "Ultrasonic Tomography":ti,ab,kw,de OR "diagnostic ultrasonic examination":ti,ab,kw,de OR "diagnostic ultrasonic imaging":ti,ab,kw,de OR "diagnostic ultrasonic method":ti,ab,kw,de OR "doptone":ti,ab,kw,de OR "duplex echography":ti,ab,kw,de OR "echogram":ti,ab,kw,de OR "echographic evaluation":ti,ab,kw,de OR "echoscopy":ti,ab,kw,de OR "echosound":ti,ab,kw,de OR "high resolution echography":ti,ab,kw,de OR "scanning, ultrasonic":ti,ab,kw,de OR "sonogram":ti,ab,kw,de OR "sonographic examination":ti,ab,kw,de OR "sonographic screening":ti,ab,kw,de OR "sonography":ti,ab,kw,de OR "ultrasonic detection":ti,ab,kw,de OR "ultrasonic echo":ti,ab,kw,de OR "ultrasonic examination":ti,ab,kw,de OR "ultrasonic scanning":ti,ab,kw,de OR "ultrasonic scintillation":ti,ab,kw,de OR "ultrasonogram":ti,ab,kw,de OR "ultrasonographic examination":ti,ab,kw,de OR "ultrasonographic screening":ti,ab,kw,de OR "ultrasound diagnosis":ti,ab,kw,de OR "ultrasound scanning":ti,ab,kw,de
	#9	"biopsy"/exp
	#10	"Biopsy":ti,ab,kw,de OR "Biopsies":ti,ab,kw,de OR "bioptic diagnosis":ti,ab,kw,de OR "bioptical diagnosis":ti,ab,kw,de OR "rebiopsy":ti,ab,kw,de
	#11	"fine needle aspiration biopsy"/exp
	#12	"Biopsy, Fine-Needle":ti,ab,kw,de OR "Biopsies, Fine-Needle":ti,ab,kw,de OR "Biopsy, Fine Needle":ti,ab,kw,de OR "Fine-Needle Biopsies":ti,ab,kw,de OR "Fine-Needle Biopsy":ti,ab,kw,de OR "Fine Needle Biopsy":ti,ab,kw,de OR "Biopsies, Fine Needle":ti,ab,kw,de OR "Fine Needle Biopsies":ti,ab,kw,de OR "Needle Biopsies, Fine":ti,ab,kw,de OR "Needle Biopsy, Fine":ti,ab,kw,de OR "Aspiration Biopsy, Fine-Needle":ti,ab,kw,de OR "Aspiration Biopsies, Fine-Needle":ti,ab,kw,de OR "Aspiration Biopsy, Fine Needle":ti,ab,kw,de OR "Biopsies, Fine-Needle Aspiration":ti,ab,kw,de OR "Biopsy, Fine-Needle Aspiration":ti,ab,kw,de OR "Fine-Needle Aspiration Biopsies":ti,ab,kw,de OR "Fine-Needle Aspiration Biopsy":ti,ab,kw,de OR "Fine-Needle Aspiration":ti,ab,kw,de OR "Aspiration, Fine-Needle":ti,ab,kw,de OR "Aspirations, Fine-Needle":ti,ab,kw,de OR "Fine Needle Aspiration":ti,ab,kw,de OR "Fine-Needle Aspirations":ti,ab,kw,de OR "fine needle aspiration biopsy":ti,ab,kw,de OR "fine core needle biopsy":ti,ab,kw,de OR "fine needle aspiration cytology":ti,ab,kw,de OR "fine needle aspirations":ti,ab,kw,de OR "FNA (fine needle aspiration)":ti,ab,kw,de OR "FNAB (fine needle aspiration biopsy)":ti,ab,kw,de OR "FNAC (fine needle aspiration cytology)":ti,ab,kw,de OR "needle aspiration":ti,ab,kw,de
	#13	#7 OR #8 OR #9 OR #10 OR #11 OR #12
	#14	"risk assessment"/exp

	#15	"Risk Assessment":ti,ab,kw,de OR "Risk Assessments":ti,ab,kw,de OR "Health Risk Assessment":ti,ab,kw,de OR "Assessment, Health Risk":ti,ab,kw,de OR "Health Risk Assessments":ti,ab,kw,de OR "Risk Assessment, Health":ti,ab,kw,de OR "Assessment, Risk":ti,ab,kw,de OR "Risks and Benefits":ti,ab,kw,de OR "Benefits and Risks":ti,ab,kw,de OR "Risk Analysis":ti,ab,kw,de OR "Analysis, Risk":ti,ab,kw,de OR "Risk Analyses":ti,ab,kw,de OR "Benefit-Risk Assessment":ti,ab,kw,de OR "Assessment, Benefit-Risk":ti,ab,kw,de OR "Benefit Risk Assessment":ti,ab,kw,de OR "Benefit-Risk Assessments":ti,ab,kw,de OR "Risk-Benefit Assessment":ti,ab,kw,de OR "Assessment, Risk-Benefit":ti,ab,kw,de OR "Risk Benefit Assessment":ti,ab,kw,de OR "Risk-Benefit Assessments":ti,ab,kw,de OR "risk stratification":ti,ab,kw,de OR "assessment, safety":ti,ab,kw,de OR "risk adjustment":ti,ab,kw,de OR "risk evaluation":ti,ab,kw,de OR "safety assessment":ti,ab,kw,de
	#16	"Korean Thyroid Association":ti,ab,kw,de OR "Korean Society of Thyroid Radiology":ti,ab,kw,de OR "American Thyroid Association":ti,ab,kw,de OR "American College of Radiology":ti,ab,kw,de OR "European Thyroid Association":ti,ab,kw,de OR "Society of Radiologists":ti,ab,kw,de OR "KTA":ti,ab,kw,de OR "KSThR":ti,ab,kw,de OR "ATA":ti,ab,kw,de OR "ACR":ti,ab,kw,de OR "ETA":ti,ab,kw,de OR "SRU":ti,ab,kw,de OR "thyroid imaging reporting and data system":ti,ab,kw,de OR "TIRADS":ti,ab,kw,de OR "TI-RADS":ti,ab,kw,de OR "Kwak-TIRADS":ti,ab,kw,de OR "K-TIRADS":ti,ab,kw,de OR "ACR-TIRADS":ti,ab,kw,de OR "EU-TIRADS":ti,ab,kw,de
	#17	#14 OR #15 OR #16
	#18	#13 AND #17
	#19	#6 AND #18
	#20	#19 NOT ("animal"/de NOT ("animal"/de AND "human"/exp))

Database	Search	Search terms
Cochrane Library	#1	[mh "Thyroid Neoplasms"]
	#2	"Thyroid Neoplasms":ti,ab,kw OR "Neoplasm, Thyroid":ti,ab,kw OR "Thyroid Neoplasm":ti,ab,kw OR "Neoplasms, Thyroid":ti,ab,kw OR "Thyroid Carcinoma":ti,ab,kw OR "Carcinoma, Thyroid":ti,ab,kw OR "Carcinomas, Thyroid":ti,ab,kw OR "Thyroid Carcinomas":ti,ab,kw OR "Cancer of Thyroid":ti,ab,kw OR "Thyroid Cancers":ti,ab,kw OR "Thyroid Cancer":ti,ab,kw OR "Cancer, Thyroid":ti,ab,kw OR "Cancers, Thyroid":ti,ab,kw OR "Cancer of the Thyroid":ti,ab,kw OR "Thyroid Adenoma":ti,ab,kw OR "Adenoma, Thyroid":ti,ab,kw OR "Adenomas, Thyroid":ti,ab,kw OR "Thyroid Adenomas":ti,ab,kw OR "thyroid tumor":ti,ab,kw OR "neoplasm of thyroid gland":ti,ab,kw OR "neoplastic thyroid":ti,ab,kw OR "neoplastic thyroid gland":ti,ab,kw OR "thyroid gland neoplasia":ti,ab,kw OR "thyroid gland neoplasm":ti,ab,kw OR "thyroid gland tumor":ti,ab,kw OR "thyroid gland tumour":ti,ab,kw OR "thyroid neoplasia":ti,ab,kw OR "thyroid tumorigenesis":ti,ab,kw OR "thyroid tumour":ti,ab,kw OR "thyroidal tumor":ti,ab,kw OR "thyroidal tumour":ti,ab,kw OR "tumor of thyroid":ti,ab,kw OR "tumor of thyroid gland":ti,ab,kw OR "tumor, thyroid gland":ti,ab,kw OR "tumour of thyroid":ti,ab,kw OR "tumour of thyroid gland":ti,ab,kw OR "tumour, thyroid gland":ti,ab,kw
	#3	[mh "Thyroid Nodule"]
	#4	"Thyroid Nodule":ti,ab,kw OR "Nodule, Thyroid":ti,ab,kw OR "Nodules, Thyroid":ti,ab,kw OR "Thyroid Nodules":ti,ab,kw OR "colloid nodule (thyroid)":ti,ab,kw OR "colloidal nodule (thyroid)":ti,ab,kw OR "nodule of the thyroid gland":ti,ab,kw OR "nodule of thyroid gland":ti,ab,kw OR "nodule, thyroid gland":ti,ab,kw OR "thyroid colloid nodule":ti,ab,kw OR "thyroid colloidal nodule":ti,ab,kw OR "thyroid gland nodule":ti,ab,kw OR "thyroid solitary nodule":ti,ab,kw OR "thyroidal gland nodule":ti,ab,kw OR "thyroidal nodule":ti,ab,kw OR "thyroideal nodule":ti,ab,kw
	#5	"thyroid mass":ti,ab,kw
	#6	#1 OR #2 OR #3 OR #4 OR #5
	#7	[mh "Ultrasonography"]

	#8	"ultrasound":ti,ab,kw OR "Ultrasonography":ti,ab,kw OR "Diagnostic Ultrasound":ti,ab,kw OR "Diagnostic Ultrasounds":ti,ab,kw OR "Ultrasound, Diagnostic":ti,ab,kw OR "Ultrasounds, Diagnostic":ti,ab,kw OR "Ultrasound Imaging":ti,ab,kw OR "Imaging, Ultrasound":ti,ab,kw OR "Imagings, Ultrasound":ti,ab,kw OR "Echotomography":ti,ab,kw OR "Ultrasonic Imaging":ti,ab,kw OR "Imaging, Ultrasonic":ti,ab,kw OR "Sonography, Medical":ti,ab,kw OR "Medical Sonography":ti,ab,kw OR "Ultrasonographic Imaging":ti,ab,kw OR "Imaging, Ultrasonographic":ti,ab,kw OR "Imagings, Ultrasonographic":ti,ab,kw OR "Ultrasonographic Imagings":ti,ab,kw OR "Echography":ti,ab,kw OR "Diagnosis, Ultrasonic":ti,ab,kw OR "Diagnoses, Ultrasonic":ti,ab,kw OR "Ultrasonic Diagnoses":ti,ab,kw OR "Ultrasonic Diagnosis":ti,ab,kw OR "Echotomography, Computer":ti,ab,kw OR "Computer Echotomography":ti,ab,kw OR "Tomography, Ultrasonic":ti,ab,kw OR "Ultrasonic Tomography":ti,ab,kw OR "diagnostic ultrasonic examination":ti,ab,kw OR "diagnostic ultrasonic imaging":ti,ab,kw OR "diagnostic ultrasonic method":ti,ab,kw OR "doptone":ti,ab,kw OR "duplex echography":ti,ab,kw OR "echogram":ti,ab,kw OR "echographic evaluation":ti,ab,kw OR "echoscopy":ti,ab,kw OR "echosound":ti,ab,kw OR "high resolution echography":ti,ab,kw OR "scanning, ultrasonic":ti,ab,kw OR "sonogram":ti,ab,kw OR "sonographic examination":ti,ab,kw OR "sonographic screening":ti,ab,kw OR "sonography":ti,ab,kw OR "ultrasonic detection":ti,ab,kw OR "ultrasonic echo":ti,ab,kw OR "ultrasonic examination":ti,ab,kw OR "ultrasonic scanning":ti,ab,kw OR "ultrasonic scintillation":ti,ab,kw OR "ultrasonogram":ti,ab,kw OR "ultrasonographic examination":ti,ab,kw OR "ultrasonographic screening":ti,ab,kw OR "ultrasound diagnosis":ti,ab,kw OR "ultrasound scanning":ti,ab,kw
	#9	[mh "Biopsy"]
	#10	"Biopsy":ti,ab,kw OR "Biopsies":ti,ab,kw OR "bioptic diagnosis":ti,ab,kw OR "bioptical diagnosis":ti,ab,kw OR "rebiopsy":ti,ab,kw
	#11	[mh "Biopsy, Fine-Needle"]
	#12	"Biopsy, Fine-Needle":ti,ab,kw OR "Biopsies, Fine-Needle":ti,ab,kw OR "Biopsy, Fine Needle":ti,ab,kw OR "Fine-Needle Biopsies":ti,ab,kw OR "Fine-Needle Biopsy":ti,ab,kw OR "Fine Needle Biopsy":ti,ab,kw OR "Biopsies, Fine Needle":ti,ab,kw OR "Fine Needle Biopsies":ti,ab,kw OR "Needle Biopsies, Fine":ti,ab,kw OR "Needle Biopsy, Fine":ti,ab,kw OR "Aspiration Biopsy, Fine-Needle":ti,ab,kw OR "Aspiration Biopsies, Fine-Needle":ti,ab,kw OR "Aspiration Biopsy, Fine Needle":ti,ab,kw OR "Biopsies, Fine-Needle Aspiration":ti,ab,kw OR "Biopsy, Fine-Needle Aspiration":ti,ab,kw OR "Fine-Needle Aspiration Biopsies":ti,ab,kw OR "Fine-Needle Aspiration Biopsy":ti,ab,kw OR "Fine-Needle Aspiration":ti,ab,kw OR "Aspiration, Fine-Needle":ti,ab,kw OR "Aspirations, Fine-Needle":ti,ab,kw OR "Fine Needle Aspiration":ti,ab,kw OR "Fine-Needle Aspirations":ti,ab,kw OR "fine needle aspiration biopsy":ti,ab,kw OR "fine core needle biopsy":ti,ab,kw OR "fine needle aspiration cytology":ti,ab,kw OR "fine needle aspirations":ti,ab,kw OR "FNA (fine needle aspiration)":ti,ab,kw OR "FNAB (fine needle aspiration biopsy)":ti,ab,kw OR "FNAC (fine needle aspiration cytology)":ti,ab,kw OR "needle aspiration":ti,ab,kw
	#13	#7 OR #8 OR #9 OR #10 OR #11 OR #12
	#14	[mh "Risk Assessment"]
	#15	"Risk Assessment":ti,ab,kw OR "Risk Assessments":ti,ab,kw OR "Health Risk Assessment":ti,ab,kw OR "Assessment, Health Risk":ti,ab,kw OR "Health Risk Assessments":ti,ab,kw OR "Risk Assessment, Health":ti,ab,kw OR "Assessment, Risk":ti,ab,kw OR "Risks and Benefits":ti,ab,kw OR "Benefits and Risks":ti,ab,kw OR "Risk Analysis":ti,ab,kw OR "Analysis, Risk":ti,ab,kw OR "Risk Analyses":ti,ab,kw OR "Benefit-Risk Assessment":ti,ab,kw OR "Assessment, Benefit-Risk":ti,ab,kw OR "Benefit Risk Assessment":ti,ab,kw OR "Benefit-Risk Assessments":ti,ab,kw OR "Risk-Benefit Assessment":ti,ab,kw OR "Assessment, Risk-Benefit":ti,ab,kw OR "Risk Benefit Assessment":ti,ab,kw OR "Risk-Benefit Assessments":ti,ab,kw OR "risk stratification":ti,ab,kw OR "assessment, safety":ti,ab,kw OR "risk adjustment":ti,ab,kw OR "risk evaluation":ti,ab,kw OR "safety assessment":ti,ab,kw
	#16	"Korean Thyroid Association":ti,ab,kw OR "Korean Society of Thyroid Radiology":ti,ab,kw OR "American Thyroid Association":ti,ab,kw OR "American College of Radiology":ti,ab,kw OR "European Thyroid Association":ti,ab,kw OR "Society of Radiologists":ti,ab,kw OR "KTA":ti,ab,kw OR "KSThR":ti,ab,kw OR "ATA":ti,ab,kw OR "ACR":ti,ab,kw OR "ETA":ti,ab,kw OR "SRU":ti,ab,kw OR "thyroid imaging reporting and data system":ti,ab,kw OR "TIRADS":ti,ab,kw OR "TI-RADS":ti,ab,kw OR "Kwak-TIRADS":ti,ab,kw OR "K-TIRADS":ti,ab,kw OR "ACR-TIRADS":ti,ab,kw OR "EU-TIRADS":ti,ab,kw
	#17	#14 OR #15 OR #16
	#18	#13 AND #17

	#19	#6 AND #18

DB	Search	Search terms
Web of Science	#1	TS=("Thyroid Neoplasms" OR "Neoplasm, Thyroid" OR "Thyroid Neoplasm" OR "Neoplasms, Thyroid" OR "Thyroid Carcinoma" OR "Carcinoma, Thyroid" OR "Carcinomas, Thyroid" OR "Thyroid Carcinomas" OR "Cancer of Thyroid" OR "Thyroid Cancers" OR "Thyroid Cancer" OR "Cancer, Thyroid" OR "Cancers, Thyroid" OR "Cancer of the Thyroid" OR "Thyroid Adenoma" OR "Adenoma, Thyroid" OR "Adenomas, Thyroid" OR "Thyroid Adenomas" OR "thyroid tumor" OR "neoplasm of thyroid gland" OR "neoplastic thyroid" OR "neoplastic thyroid gland" OR "thyroid gland neoplasia" OR "thyroid gland neoplasm" OR "thyroid gland tumor" OR "thyroid gland tumour" OR "thyroid neoplasia" OR "thyroid tumorigenesis" OR "thyroid tumour" OR "thyroidal tumor" OR "thyroidal tumour" OR "tumor of thyroid" OR "tumor of thyroid gland" OR "tumor, thyroid gland" OR "tumour of thyroid" OR "tumour of thyroid gland" OR "tumour, thyroid gland")
	#2	TS=("Thyroid Nodule" OR "Nodule, Thyroid" OR "Nodules, Thyroid" OR "Thyroid Nodules" OR "colloid nodule (thyroid)" OR "colloidal nodule (thyroid)" OR "nodule of the thyroid gland" OR "nodule of thyroid gland" OR "nodule, thyroid gland" OR "thyroid colloid nodule" OR "thyroid colloidal nodule" OR "thyroid gland nodule" OR "thyroid solitary nodule" OR "thyroidal gland nodule" OR "thyroidal nodule" OR "thyroideal nodule")
	#3	TS=("thyroid mass")
	#4	#1 OR #2 OR #3
	#5	TS=("ultrasound" OR "Ultrasonography" OR "Diagnostic Ultrasound" OR "Diagnostic Ultrasounds" OR "Ultrasound, Diagnostic" OR "Ultrasounds, Diagnostic" OR "Ultrasound Imaging" OR "Imaging, Ultrasound" OR "Imagings, Ultrasound" OR "Echotomography" OR "Ultrasonic Imaging" OR "Imaging, Ultrasonic" OR "Sonography, Medical" OR "Medical Sonography" OR "Ultrasonographic Imaging" OR "Imaging, Ultrasonographic" OR "Imagings, Ultrasonographic" OR "Ultrasonographic Imagings" OR "Echography" OR "Diagnosis, Ultrasonic" OR "Diagnoses, Ultrasonic" OR "Ultrasonic Diagnoses" OR "Ultrasonic Diagnosis" OR "Echotomography, Computer" OR "Computer Echotomography" OR "Tomography, Ultrasonic" OR "Ultrasonic Tomography" OR "diagnostic ultrasonic examination" OR "diagnostic ultrasonic imaging" OR "diagnostic ultrasonic method" OR "doptone" OR "duplex echography" OR "echogram" OR "echographic evaluation" OR "echoscopy" OR "echosound" OR "high resolution echography" OR "scanning, ultrasonic" OR "sonogram" OR "sonographic examination" OR "sonographic screening" OR "sonography" OR "ultrasonic detection" OR "ultrasonic echo" OR "ultrasonic examination" OR "ultrasonic scanning" OR "ultrasonic scintillation" OR "ultrasonogram" OR "ultrasonographic examination" OR "ultrasonographic screening" OR "ultrasound diagnosis" OR "ultrasound scanning")
	#6	TS=("Biopsy" OR "Biopsies" OR "bioptic diagnosis" OR "bioptical diagnosis" OR "rebiopsy")
	#7	TS=("Biopsy, Fine-Needle" OR "Biopsies, Fine-Needle" OR "Biopsy, Fine Needle" OR "Fine-Needle Biopsies" OR "Fine-Needle Biopsy" OR "Fine Needle Biopsy" OR "Biopsies, Fine Needle" OR "Fine Needle Biopsies" OR "Needle Biopsies, Fine" OR "Needle Biopsy, Fine" OR "Aspiration Biopsy, Fine-Needle" OR "Aspiration Biopsies, Fine-Needle" OR "Aspiration Biopsy, Fine Needle" OR "Biopsies, Fine-Needle Aspiration" OR "Biopsy, Fine-Needle Aspiration" OR "Fine-Needle Aspiration Biopsies" OR "Fine-Needle Aspiration Biopsy" OR "Fine-Needle Aspiration" OR "Aspiration, Fine-Needle" OR "Aspirations, Fine-Needle" OR "Fine Needle Aspiration" OR "Fine-Needle Aspirations" OR "fine needle aspiration biopsy" OR "fine core needle biopsy" OR "fine needle aspiration cytology" OR "fine needle aspirations" OR "FNA (fine needle aspiration)" OR "FNAB (fine needle aspiration biopsy)" OR "FNAC (fine needle aspiration cytology)" OR "needle aspiration")
	#8	#5 OR #6 OR #7
	#9	TS=("Risk Assessment" OR "Risk Assessments" OR "Health Risk Assessment" OR "Assessment, Health Risk" OR "Health Risk Assessments" OR "Risk Assessment, Health" OR "Assessment, Risk" OR "Risks and Benefits" OR "Benefits and Risks" OR "Risk Analysis" OR "Analysis, Risk" OR "Risk Analyses" OR "Benefit-Risk Assessment" OR "Assessment, Benefit-Risk" OR "Benefit Risk Assessment" OR "Benefit-Risk Assessments" OR "Risk-Benefit Assessment" OR "Assessment, Risk-Benefit" OR "Risk Benefit Assessment" OR "Risk-Benefit Assessments" OR "risk stratification" OR "assessment, safety" OR "risk adjustment" OR "risk evaluation" OR "safety assessment")

	#10	TS=("Korean Thyroid Association" OR "Korean Society of Thyroid Radiology" OR "American Thyroid Association" OR "American College of Radiology" OR "European Thyroid Association" OR "American Association of Clinical Endocrinologists" OR "Society of Radiologists" OR "KTA" OR "KSThR" OR "ATA" OR "ACR" OR "ETA" OR "SRU" OR "thyroid imaging reporting and data system" OR "TIRADS" OR "TI-RADS" OR "Kwak-TIRADS" OR "K-TIRADS" OR "ACR-TIRADS" OR "EU-TIRADS")
	#11	#9 OR #10
	#12	#8 AND #11
	#13	#4 AND #12

<table>
<tr><th>Database</th><th>Search</th><th>Search terms</th></tr>
<tr><td rowspan="4">Google Scholar</td><td>#1</td><td>"Thyroid Neoplasm"|"Thyroid Neoplasms"|"Thyroid Cancers"|"Thyroid Cancer"|"Thyroid Nodule"|"Thyroid Nodules"|"thyroid mass"</td></tr>
<tr><td>#2</td><td>"Ultrasonography"|"ultrasound"|"Biopsy"|"Biopsies"|"Fine-Needle"</td></tr>
<tr><td>#3</td><td>"Risk Assessment"|"risk stratification"|KTA|KSThR|ATA|ACR|ETA|AACE|ACE|AME|SRU|TIRADS|TI-RADS</td></tr>
<tr><td>#4</td><td>(Thyroid Neoplasm|Thyroid Cancer|Thyroid Nodule|"thyroid mass") AND (("Ultrasonography"|"ultrasound"|"Biopsy"|"Biopsies"|"Fine-Needle") AND ("Risk Assessment"|"risk stratification"|KTA|KSThR|ATA|ACR|ETA|SRU|TIRADS|TI-RADS))</td></tr>
</table>

Supplementary Table S2. Study characteristics

Study	Year	Type	Patients Number	Age	Sex (F/M)	Nationality	Mean nodule′s size	Tumor size	per specimen	Cutoff value	Risk Stratification Systems	Reference test
Chng 2015	2015	retrospective study	144	49.6 ± 15.7 yrs	110/34	UK	3.2 ± 1.5 cm		144	4a	TIRADS (Kwak)	only cytology
Yoon 2015	2015	Retrospective	1241	50.8 yrs	NA	Korea	18.8 ± 10.7 mm	>1.0 cm	1293	4a	TIRADS (Kwak), ATA	cytological and histological diagnoses
Zhang 2015	2015	prospective study	2921	NA	NA	China	NA	all	3980	4a	TIRADS (Kwak)	cytological and histological diagnoses
Srinivas 2016	2016	prospective study	365	33.1 yrs (range: 18-68)	343/22	India	14.2 mm (range: 3-59)		365	4a	TIRADS (Kwak)	cytologically or pathologically confirm
Ha 2017	2017	Prospective multicenter study	750	49.2 yrs (range: 9-81)	594/156	Korea	1.5 ± 1.1 cm (0.5-10.0 cm)	> 5 mm	902	Low suspicion	TIRADS (Korea)	cytologically or pathologically confirm
Mao 2017	2017	retrospective study of	121	54±13 yrs (range: 25-76)	103/18	China	9.1±4.6 mm (5-25)		121	4a	TIRADS (Kwak)	cytologically or pathologically confirm
Ting 2017	2017	Retrospective	734	46.8 ± 13.1 yrs	578/156	China	17.7 ± 12.8mm	<10 mm	962	High suspicion	ATA	cytologically or pathologically confirm
Trimboli 2017	2017	Retrospective	173	50 ± 14 yrs	127/46	Switzerland	2.5 cm	>1.0 cm (n=157)	173	Low suspicion	ATA	postoperative histology
Yoon 2017	2017	retrospectively review	4585	46.7 ± 12.7 yrs	3836/749	Korea	12.7 ± 2.6 mm	1?2 cm	4696	4a	TIRADS (Kwak), ATA	cytological and histological diagnoses
Bae 2018	2018	Prospective study	190	49.7 ± 11.3 (range: 20-83)	183/55	Korea	2.2 ± 1.2 cm (range: 1.0-6.0)	≥ 10 mm	201	Intermediate suspicion	TIRADS (Korea)	cytologically or pathologically confirm
Chng 2018	2018	Retrospective	150	54.4 ± 12.4 yrs	120/30	Singapore	3.1 ± 1.5 cm	> 1 cm	167	4a	TIRADS (Kwak)	Surgery
Chng 2018	2018	Retrospective	150	54.4 ± 12.4 yrs	120/30	Singapore	3.1 ± 1.5 cm	> 1 cm	167	Low suspicion	ATA	surgery
de Macedo 2018	2018	Retrospective	178	59 yrs (range: 49-66)	167/11	Brazil	24 mm (range 15-37)	> 1 cm	195	High suspicion	ATA, EU TI-RADS	cytopathologic results of the Bethesda system, and surgical findings
Ha 2018	2018	Retrospective	750	49.2 yrs (range: 9-81)	594/156	Korea	1.5 ±1.1 cm	>5mm	902	highly suspicious (TR5).	ACR TI-RADS	cytopathologic results of the Bethesda system, and surgical findings
Ha 2018	2018	Retrospective	750	49.2 yrs (range: 9-81)	594/156	Korea	1.5 ±1.1 cm	>5mm	902	High suspicion	ATA, TIRADS (Korea)	cytopathologic results of the Bethesda system, and surgical findings
Persichetti 2018	2018	Prospective observational study	789	55 ± 14 yrs	608/181	Italy	21.2 ± 13.4 mm		1100	High suspicion	ATA	cytopathologic results of the Bethesda system, and surgical findings
Ahmadi 2019	2019	retrospectively review	186	57 yrs (IQR: 44-68)	154/32	USA	2.4 cm (IQR: 1.5-3.5)		202	highly suspicious (TR5).	ACR TI-RADS, ATA	surgical pathology
Barbosa 2019	2019	retrospective observational study	139	49 ± 13 yrs	118/21	Brazil	1.7 cm		140	mildly suspicious (TR3)	ACR TI-RADS, ATA	cytopathologic results of the Bethesda system, and surgical findings
Gao 2019	2019	retrospectively review	1758	48.5 ± 12.0 yrs	1788/756	China	1.1 ± 0.7 cm		2544	4a	TIRADS (Kwak), ACR TI-RADS, ATA	surgical pathology
Gitto 2019	2019	retrospectively review	62	60 ± 12 yrs	50/12	Italy	18 ± 7 mm		62	Intermediate suspicio	TIRADS (Korea)	only cytology

										n		
Ha 2019	2019	retrospective study	3190	53.4 ± 12.2 yrs	2517/673	Korea	1.4 cm (range: 0.3-9.6)		3323	mildly suspicious (TR3)	ACR TI-RADS, ATA, TIRADS (Korea)	cytological and histological diagnoses
Ha 2019	2019	retrospective study	2747	51.4 ± 14.4 yrs	532/2111	Korea	14.4 mm (range: 2-96)		2747	Intermediate suspicion	ATA, TIRADS (Korea)	cytologically or pathologically confirm
Hong 2019	2019	retrospectively review	683	48.50 ± 11.78 yrs	568/115	Korea	1.32 ± 1.02 cm		683	High suspicion	ATA, TIRADS (Korea)	cytopathologic results of the Bethesda system, and surgical findings
Li 2019	2019	Retrospective hospital-based cohort study	93	55.21 ± 2.95 yrs	73/20	China	1.72 ± 0.18 cm		93	4a	TIRADS (Kwak), ATA	surgical pathology
Madsen Barbosa 2019	2019	retrospective observational study	139	48.6 ± 13.3 yrs (25-77)	118/21	Brazil	NA		140	mildly suspicious (TR3)	ACR TI-RADS, ATA	cytological and histological diagnoses
Phutthar ak 2019	2019	retrospective study	94	51.6 ±13.08 yrs	7/87	Thailand	2.12 cm (range: 0.46-8.0)		108	moderately suspicious (TR4)	ACR TI-RADS, EU TI-RADS	only cytology
Ruan 2019	2019	retrospective study	918	45.7 yrs (range: 14-78)	562/356	China	14.5 ± 9.4 mm		1001	highly suspicious (TR5).	ACR TI-RADS, ATA	cytopathologic results of the Bethesda system, and surgical findings
Shen 2019	2019	retrospectively review	1568	48 ± 13 yrs	1192/420	China	13.59 ± 11.01 mm	>5mm	1612	4a	TIRADS (Kwak), ACR TI-RADS, ATA, EU TI-RADS	cytopathologic results of the Bethesda system, and surgical findings
Wu 2019	2019	retrospectively review	894	NA	NA	China	NA		1000	highly suspicious (TR5).	ACR TI-RADS, ATA	cytopathologic results of the Bethesda system, and surgical findings
Wu 2019	2019	Prospective study	29	47.6 ± 15.5 yrs	NA	China	25.8 ± 13.0 mm (range: 4-62)		43	mildly suspicious (TR3)	ACR TI-RADS	surgical pathology
Xiang 2019	2019	retrospective study	505	48.9±2.2 yrs	401/114	China	1.78±1.42 cm (range: 0.16-7.1cm)	all	708	High suspicion	ATA, TIRADS (Korea)	surgical pathology
Xu 2019	2019	retrospective study	2031	47.68 ± 13.39 yrs	1616/415	China	16.63 ± 11.78 mm		2031	mildly suspicious (TR3)	ACR TI-RADS, EU TI-RADS, TIRADS (Korea)	cytologically or pathologically confirm
Yoon 2019	2019	retrospectively review	1836	55.1 yrs	1494/342	Korea	19.4 ± 9.9 mm (range: 10-90)	≥1 cm	2274	mildly suspicious (TR3)	ACR TI-RADS, EU TI-RADS, TIRADS (Korea)	cytological and histological diagnoses
Huang 2020	2020	retrospectively review	260	58 yrs (range: 46-69)	212/48	USA	2.2 (IQR: 1.6-3.3)		361	mildly suspicious (TR3)	ACR TI-RADS, ATA	cytology diagnoses
Koc 2020	2020	retrospectively review	460	52 yrs (range: 18-84)	375/85	Turkey	NA		492	mildly suspicious (TR3)	ACR TI-RADS, ATA, EU TI-RADS	histopathology
Peng 2020 (Junior)	2020	Retrospective diagnostic study	230	44.8 ± 11.5 yrs	171/59	China	15.6 ± 10.7 mm (range: 3.0-52.0)		230	mildly suspicious (TR3)	ACR TI-RADS, ATA	cytologically or pathologically confirm
Szczepanek-Parulska 2020	2020	prospective study	88	49.4 ± 15.5 yrs (range: 17-80)	70/18	Poland	NA		133	Intermediate risk	EU TI-RADS	surgical pathology
Watkins 2020	2020	retrospectively review	212	58.5 ± 29 yrs	161/61	UK	19 ± 19.1 mm		218	mildly suspicious (TR3)	ACR TI-RADS	histopathology
Xiao 2020	2020	Retrospective diagnostic study	792	48.25 ± 12.37 yrs	632/160	China	0.9 cm (range: 0.5-1.9)	all	1940	4a	TIRADS (Kwak), ACR TI-RADS	cytologically or pathologically confirm

Yang 2020	2020	retrospectively review	92	51.1 yrs	70/22	USA	2.1 cm (range: 0.8-7.7)		92	mildly suspicious (TR3)	ACR TI-RADS, ATA, TIRADS (Korea)	surgical pathology
Yoo 2020	2020	retrospective observational study	382	47.4±12.6 yrs	297/85	Korea	1.14 ± 0.82 cm		382	mildly suspicious (TR3)	ACR TI-RADS, ATA, EU TI-RADS, TIRADS (Korea)	cytologically or pathologically confirm
Yoon 2020	2020	retrospective design	8364	50.1 ± 13.5 yrs	6598/1766	Korea	22.0±12.1 mm (range: 10-113)		8657	4a	TIRADS (Kwak), ACR TI-RADS, ATA, EU TI-RADS, TIRADS (Korea)	cytologically or pathologically confirm
Zhang 2020	2020	retrospective study	1271	51.7±12.5 yrs	986/285	China	16.4±11.6 mm	all	1271	4a	TIRADS (Kwak), ACR TI-RADS, ATA, TIRADS (Korea)	cytologically or pathologically confirm
Celletti	2021	prospective study	128	54.3 yrs (range: 18-82)	89/39	Italy	NA	all	128	Low suspicion	TIRADS (Korea)	cytologically or pathologically confirm
Chen 2021	2021	retrospective study	125	56.7 yrs	99/26	Australia	median: 2.3 cm (range: 0.1-13)		146	mildly suspicious (TR3)	ACR TI-RADS, ATA, TIRADS (Korea)	cytologically or pathologically confirm
Chung 2021	2021	retrospective study	5081	53.2 ± 12.7 yrs	4176/905	Korea	2.1 ± 1.1 cm (range: 1-10)	≥1 cm	5708	Low suspicion	TIRADS (Korea)	cytologically or pathologically confirm
da Silva 2021 (cytology)	2021	retrospectively review	314	51.56 ± 15.4 yrs	NA	Brazil	1.69 ± 1.32 cm		473	moderately suspicious (TR4)	ACR TI-RADS, EU TI-RADS	cytologically or pathologically confirm
Ha 2021	2021	retrospectively multicenter review	5081	53.2 yrs (range: 19-93)	4176/905	Korea	20.7±10.8 mm (range: 10-100)	≥1.0 cm	5708	mildly suspicious (TR3)	ACR TI-RADS, EU TI-RADS, TIRADS (Korea)	cytologically or pathologically confirm
Han 2021	2021	retrospectively multicenter review	372	49.5 yrs (range: 8-81)	289/83	Korea	17.8 mm (range: 10-73)	≥1.0 cm	454	mildly suspicious (TR3)	ACR TI-RADS, TIRADS (Korea)	cytologically or pathologically confirm
Hekimsoy 2021	2021	retrospective study	165	49.64±13.50 yrs	131/34	Turkey	15.0 mm (range: 10-29.25)		251	mildly suspicious (TR3)	ACR TI-RADS, EU TI-RADS	cytologically or pathologically confirm
Huh 2021	2021	retrospective study	1301	50.2 ± 13.6 yrs	1062/239	Korea	23.2 ± 12.6 mm (range: 10-100)		1384	4a	TIRADS (Kwak), ACR TI-RADS, ATA, EU TI-RADS	cytologically or pathologically confirm
Kang 2021	2021	retrospective study	160	51.2 ± 14.5 yrs	145/15	Korea	1.7±1.1 cm		200	mildly suspicious (TR3)	ACR TI-RADS, TIRADS (Korea)	cytologically or pathologically confirm
Na 2021	2021	retrospective study	3088	median 56 yrs (IQR: 47-64)	2497/591	Korea	1.7 cm (range: 1-10 cm)	≥1 cm	3826	mildly suspicious (TR3)	ACR TI-RADS, ATA, EU TI-RADS, TIRADS (Korea)	cytologically or pathologically confirm
Qi 2021	2021	retrospective study	884	41.83 yrs (range: 10-73)	681/203	China	19.13 mm (range: 5-64)		1096	4a	TIRADS (Kwak), ACR TI-RADS, EU TI-RADS, TIRADS (Korea)	cytologically or pathologically confirm
Scappaticcio 2021	2021	retrospective analysis	36	15 yrs (range: 11-17)	26/10	Switzerland	10 mm (range: 7-13)		41	mildly suspicious (TR3)	ACR TI-RADS, ATA, EU TI-RADS, TIRADS (Korea)	cytologically or pathologically confirm
Seifert 2021	2021	retrospective study	849	51 ± 14 yrs	604/249	Germany	26 ± 13 mm		1211	4c	TIRADS (Kwak), ACR TI-RADS, ATA, EU TI-RADS, TIRADS (Korea)	cytologically or pathologically confirm
Seminati 2021	2021	prospective study	448	NA	NA	Italy	NA		493	mildly suspicious (TR3)	ACR TI-RADS, EU TI-RADS	cytologically or pathologically confirm
Shi 2021	2021	retrospective study	839	54 yrs (range: 14-88)	594/245	China	22 mm (range: 14-32)		846	4a	TIRADS (Kwak), ACR TI-RADS, EU TI-RADS	cytologically or pathologically confirm
Zhang 2021	2021	retrospective study	566	47.4±13.4 yrs	442/124	China	11.4±5.8 mm (range: 5.0-36.0)		566	4a	TIRADS (Kwak), ACR TI-RADS, ATA, TIRADS (Korea)	cytologically or pathologically confirm

Zhu 2021	2021	retrospective study	1697	53.2 ± 12.7 yrs	1336/361	China	13.1±10.6 mm (range: 0.2-69)	all	2309	mildly suspicious (TR3)	ACR TI-RADS, ATA, TIRADS (Korea)	cytologically or pathologically confirm
Alqahtani 2022	2022	retrospective cohort study	50	46.26 ± 2.08 yrs	42/8	Pakistan	NA		50	mildly suspicious (TR3)	ACR TI-RADS	surgical pathology
Chen 2022	2022	retrospectively review	1978	46.41 ± 12.65 yrs	1531/351	China	11.71 ± 8.47 mm (range: 2-73)		1982	mildly suspicious (TR3)	ACR TI-RADS, TIRADS (Korea)	cytologically or pathologically confirm
Lin 2022	2022	retrospective analysis	329	43.5 ± 14.3 yrs	216/113	China	3.6 ± 1.7 cm (range: 0.6-12.1)		329	mildly suspicious (TR3)	ACR TI-RADS, ATA, EU TI-RADS, TIRADS (Korea)	surgical pathology
Qi 2022	2022	single center retrospective study	820	44.5 ± 13.4 yrs	619/201	China	13 mm (range: 11-17)		820	4a	TIRADS (Kwak), ACR TI-RADS, ATA	cytologically or pathologically confirm
Thedinger 2022	2022	Case series with prospective data collection and retrospective chart review	720	58.7 ± 0.6 yrs	538/182	USA	NA		949	mildly suspicious (TR3)	ACR TI-RADS, ATA	only cytology
Zhang 2022	2022	retrospective study	509	48.22 ± 12.31 yrs	355/154	China	1.80 cm (range: 0.80-3.40)		509	4a	TIRADS (Kwak), ACR TI-RADS	cytologically or pathologically confirm
Zhang 2022	2022	retrospective study of	585	44 yrs (range: 34-51)	487/98	China	NA		585	Intermediate suspicion	TIRADS (Korea)	surgical pathology

Supplementary Table S3. Methodological quality of the included studies.

Reference	Risk of Bias				Concerns about Application		
	Patient Selection	Index test	Reference standard	Flow and timing	Patient selection	Index test	Reference Standard
Chng 2015	Low	Low	Low	Unclear	Low	Low	Low
Yoon 2015	Low	Low	Low	Unclear	Low	Low	Low
Zhang 2015	Low	Low	Low	Unclear	Low	Low	Low
Srinivas 2016	Low	Low	Low	Low	Low	Low	Low
Ha 2017	Low	Low	Low	Unclear	Low	Low	Low
Mao 2017	Low	Low	Low	Unclear	Low	Low	Low
Ting 2017	Low	Low	Low	Low	Low	Low	Low
Trimboli 2017	Low	Low	Low	Unclear	Low	Low	Low
Yoon 2017	Low	Low	Low	Low	Low	Low	Low
Bae 2018	Low	Low	Low	Unclear	Low	Low	Low
Chng 2018	Low	Low	Low	Low	Low	Low	Low
Chng 2018	Low	Low	Low	Low	Low	Low	Low
de Macedo 2018	Low	Low	Low	Unclear	Low	Low	Low
Ha 2018	Low	Low	Low	Low	Low	Low	Low
Ha 2018	Low	Low	Low	Low	Low	Low	Low
Persichetti 2018	Low	Low	Low	Unclear	Low	Low	Low
Ahmadi 2019	Low	Low	Low	Low	Low	Low	Low
Barbosa 2019	Low	Low	Low	Unclear	Low	Low	Low
Gao 2019	Low	Low	Low	Low	Low	Low	Low
Gitto 2019	Low	Low	Low	Unclear	Low	Low	Low
Ha 2019	Low	Low	Low	Low	Low	Low	Low
Ha 2019	Low	Low	Low	Unclear	Low	Low	Low
Hong 2019	Low	Low	Low	Low	Low	Low	Low
Li 2019	Low	Low	Low	Unclear	Low	Low	Low
Madsen Barbosa 2019	Low	Low	Low	Low	Low	Low	Low
Phuttharak 2019	Low	Low	Low	Low	Low	Low	Low
Ruan 2019	Low	Low	Low	Unclear	Low	Low	Low
Shen 2019	Low	Low	Low	Low	Low	Low	Low
Wu 2019	Low	Low	Low	Low	Low	Low	Low
Wu 2019	Low	Low	Low	Unclear	Low	Low	Low
Xiang 2019	Low	Low	Low	Unclear	Low	Low	Low
Xu 2019	Low	Low	Low	Unclear	Low	Low	Low
Yoon 2019	Low	Low	Low	Unclear	Low	Low	Low
Huang 2020	Low	Low	Low	Low	Low	Low	Low
Koc 2020	Low	Low	Low	Unclear	Low	Low	Low
Peng 2020 (Junior)	Low	Low	Low	Unclear	Low	Low	Low
Szczepanek-Parulska 2020	Low	Low	Low	Low	Low	Low	Low
Watkins 2020	Low	Low	Low	Unclear	Low	Low	Low
Xiao 2020	Low	Low	Low	Low	Low	Low	Low
Yang 2020	Low	Low	Low	Unclear	Low	Low	Low
Yoo 2020	Low	Low	Low	Low	Low	Low	Low
Yoon 2020	Low	Low	Low	Unclear	Low	Low	Low
Zhang 2020	Low	Low	Low	Unclear	Low	Low	Low
Celletti	Low	Low	Low	Low	Low	Low	Low
Chen 2021	Low	Low	Low	Unclear	Low	Low	Low
Chung 2021	Low	Low	Low	Low	Low	Low	Low
da Silva 2021 (cytology)	Low	Low	Low	Unclear	Low	Low	Low
Ha 2021	Low	Low	Low	Low	Low	Low	Low
Han 2021	Low	Low	Low	Low	Low	Low	Low
Hekimsoy 2021	Low	Low	Low	Unclear	Low	Low	Low
Huh 2021	Low	Low	Low	Low	Low	Low	Low
Kang 2021	Low	Low	Low	Low	Low	Low	Low
Na 2021	Low	Low	Low	Unclear	Low	Low	Low
Qi 2021	Low	Low	Low	Low	Low	Low	Low
Scappaticcio 2021	Low	Low	Low	Unclear	Low	Low	Low
Seifert 2021	Low	Low	Low	Unclear	Low	Low	Low
Seminati 2021	Low	Low	Low	Low	Low	Low	Low
Shi 2021	Low	Low	Low	Unclear	Low	Low	Low
Zhang 2021	Low	Low	Low	Low	Low	Low	Low
Zhu 2021	Low	Low	Low	Unclear	Low	Low	Low
Alqahtani 2022	Low	Low	Low	Low	Low	Low	Low
Chen 2022	Low	Low	Low	Low	Low	Low	Low

Lin 2022	Low	Low	Low	Unclear	Low	Low	Low
Qi 2022	Low	Low	Low	Low	Low	Low	Low
Thedinger 2022	Low	Low	Low	Unclear	Low	Low	Low
Zhang 2022	Low	Low	Low	Unclear	Low	Low	Low
Zhang 2022	Low	Low	Low	Unclear	Low	Low	Low

Supplementary Table S4. Brief description of the Risk Stratification Systems

TIRADS	Society	Year	Description
K-TIRADS	Korean Society of Thyroid Radiology	2016	Four categories from low to high suspicion<br>Classification according to suspicious US features based on echogenicity
ACR-TIRADS	American College of Radiology	2017	Five categories from benign to highly suspicious<br>US Features with Point Values
ATA	American Thyroid Association	2015	Five categories from benign to high suspicion<br>Atlas of sonographic features for classification of nodules
EU- TIRADS	European Thyroid Association	2017	Five categories from normal to high risk<br>High suspicion if any of the four characteristics are present
Kwak-TIRADS	Kwak et al.	2011	Four categories according to the number of suspicious US features

Supplementary Figure S1. Begg's funnel plot for K-TIRAD.

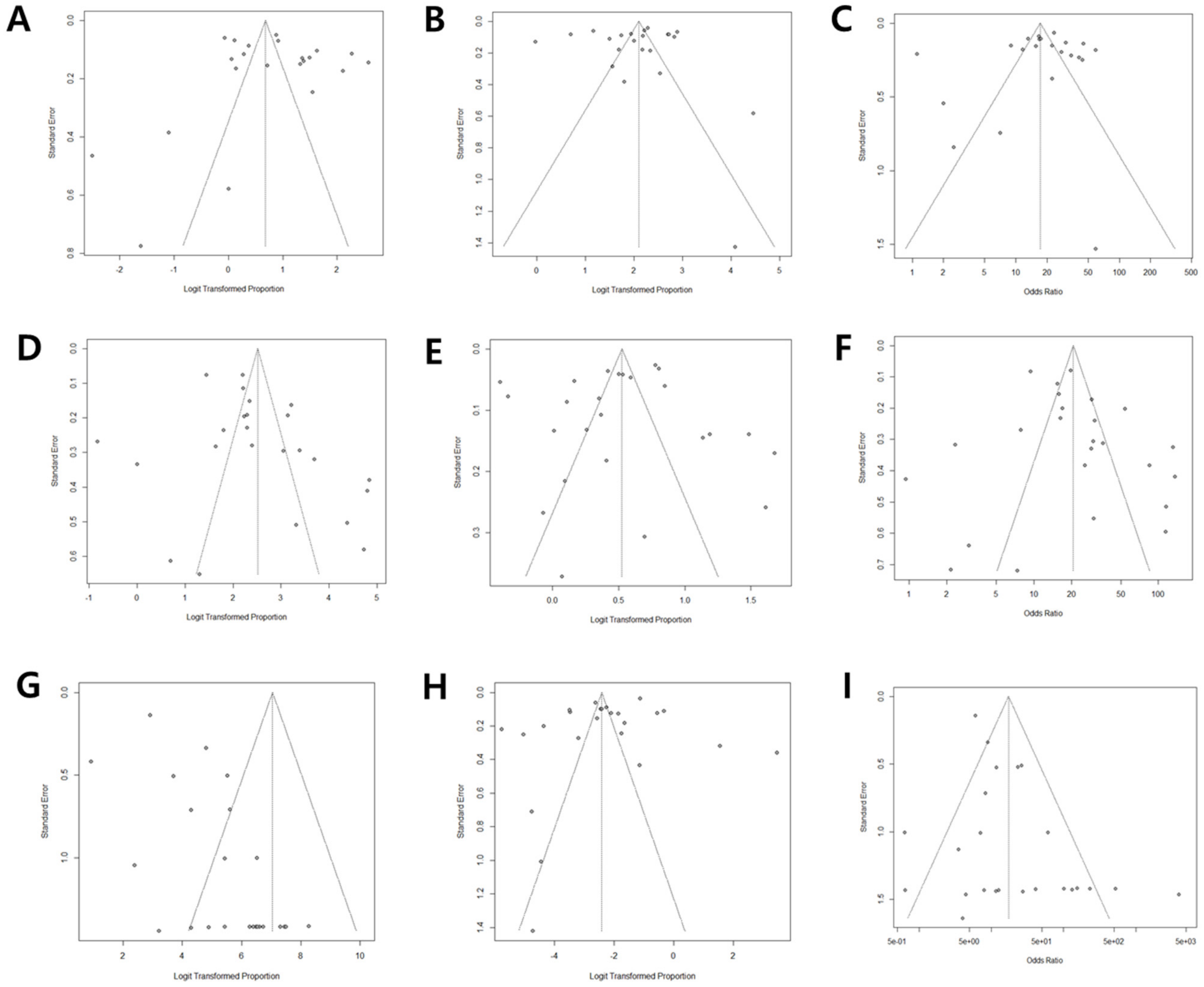

A. High K-TIRAD, sensitivity
B. High K-TIRAD, specificity
C. High K-TIRAD, DOR
D. Intermediate K-TIRAD, sensitivity
E. Intermediate K-TIRAD, specificity
F. Intermediate K-TIRAD, DOR
G. Low K-TIRAD, sensitivity
H. Low K-TIRAD, specificity
I. Low K-TIRAD, DOR

Supplementary Figure S2. Begg's funnel plot for ATA

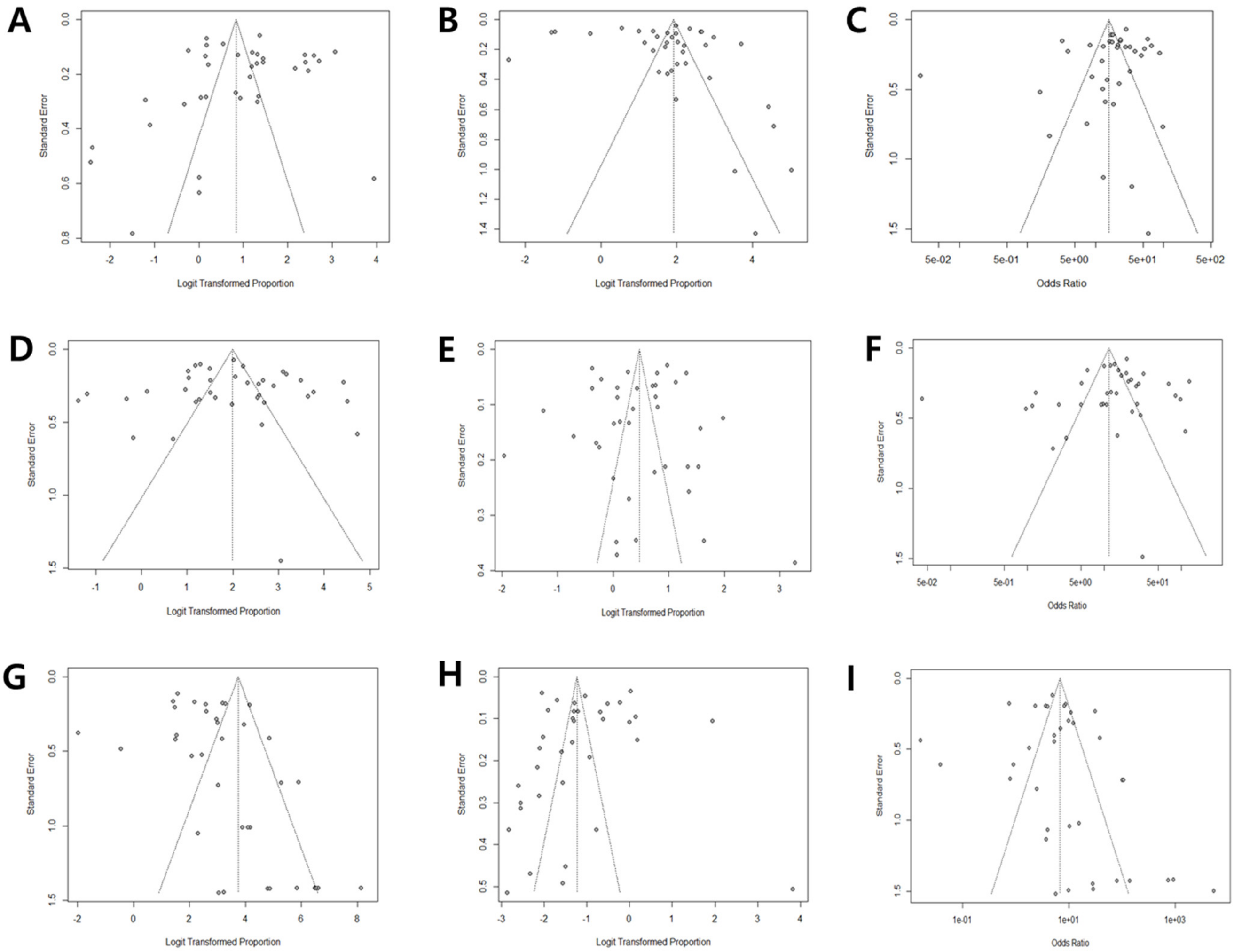

A. High ATA, sensitivity
B. High ATA, specificity
C. High ATA, DOR
D. Intermediate ATA, sensitivity
E. Intermediate ATA, specificity
F. Intermediate ATA, DOR
G. Low ATA, sensitivity
H. Low ATA, specificity
I. Low ATA, DOR

Supplementary Figure S3. Begg's funnel plot for ACR

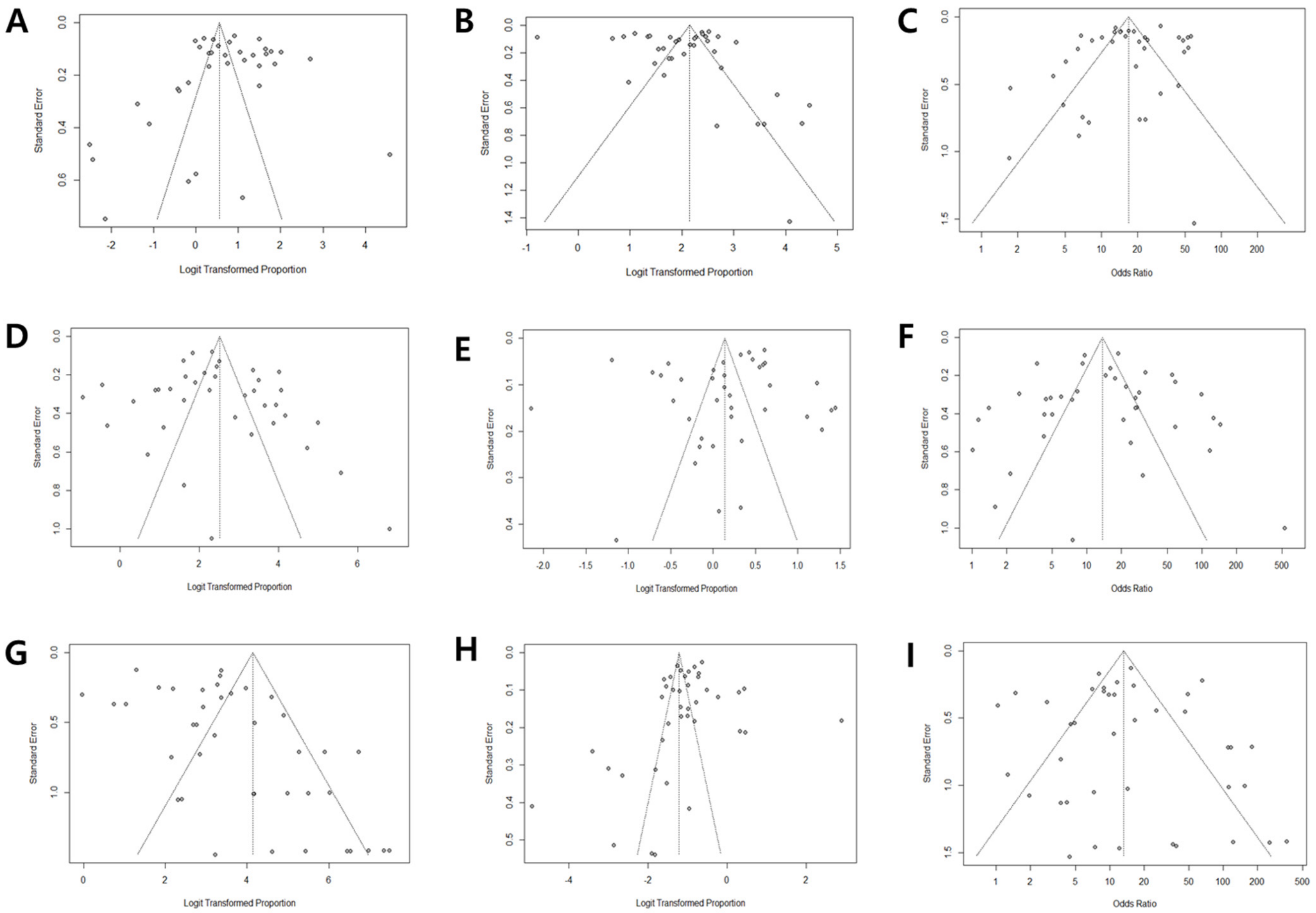

A. High ACR, sensitivity
B. High ACR, specificity
C. High ACR, DOR
D. Intermediate ACR, sensitivity
E. Intermediate ACR, specificity
F. Intermediate ACR, DOR
G. Low ACR, sensitivity
H. Low ACR, specificity
I. Low ACR, DOR

Supplementary Figure S4. Begg’s funnel plot for EU

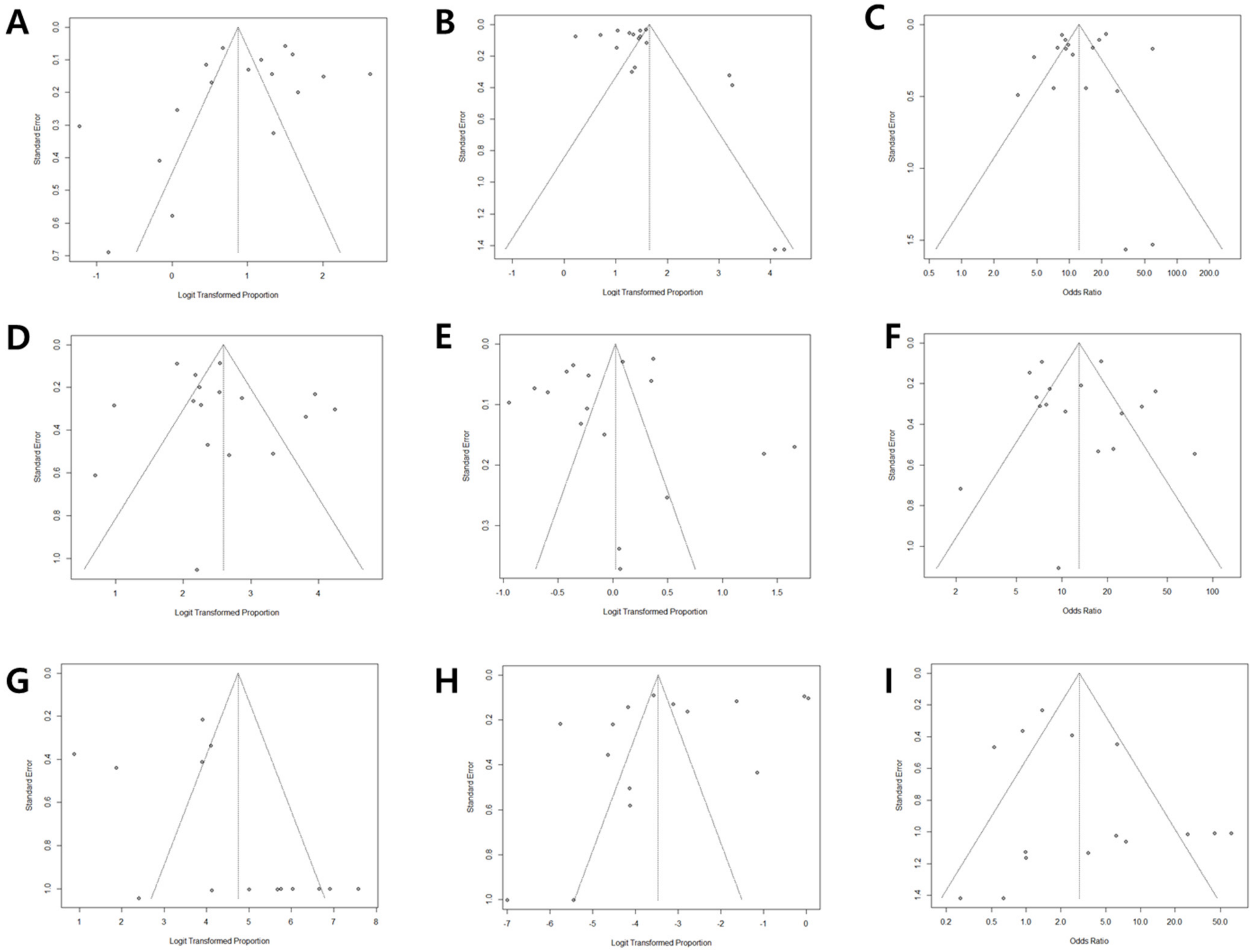

A. High ATA, sensitivity
B. High ATA, specificity
C. High ATA, DOR
D. Intermediate ATA, sensitivity
E. Intermediate ATA, specificity
F. Intermediate ATA, DOR
G. Low ATA, sensitivity
H. Low ATA, specificity
I. Low ATA, DOR

Supplementary Figure S5. Begg's funnel plot for Kwak-TIRAD

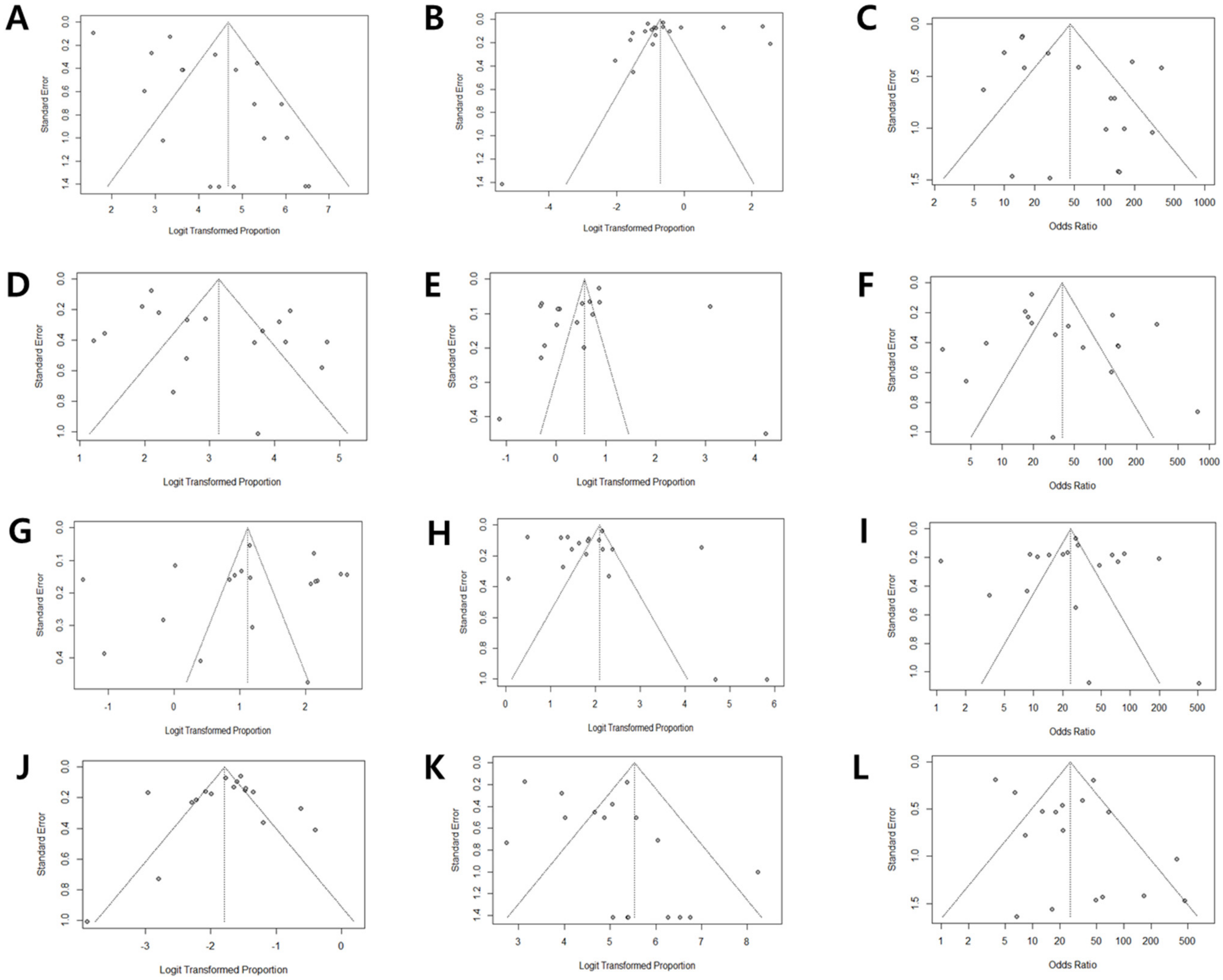

A. 4a, sensitivity
B. 4a, specificity
C. 4a, DOR
D. 4b, sensitivity
E. 4b, specificity
F. 4b, DOR
G. 4c, sensitivity
H. 4c, specificity
I. 4c, DOR
J. 5, sensitivity
K. 5, specificity
L. 5, DOR

Supplementary Figure S6. Forest plot of the diagnostic odds ratio for ACR

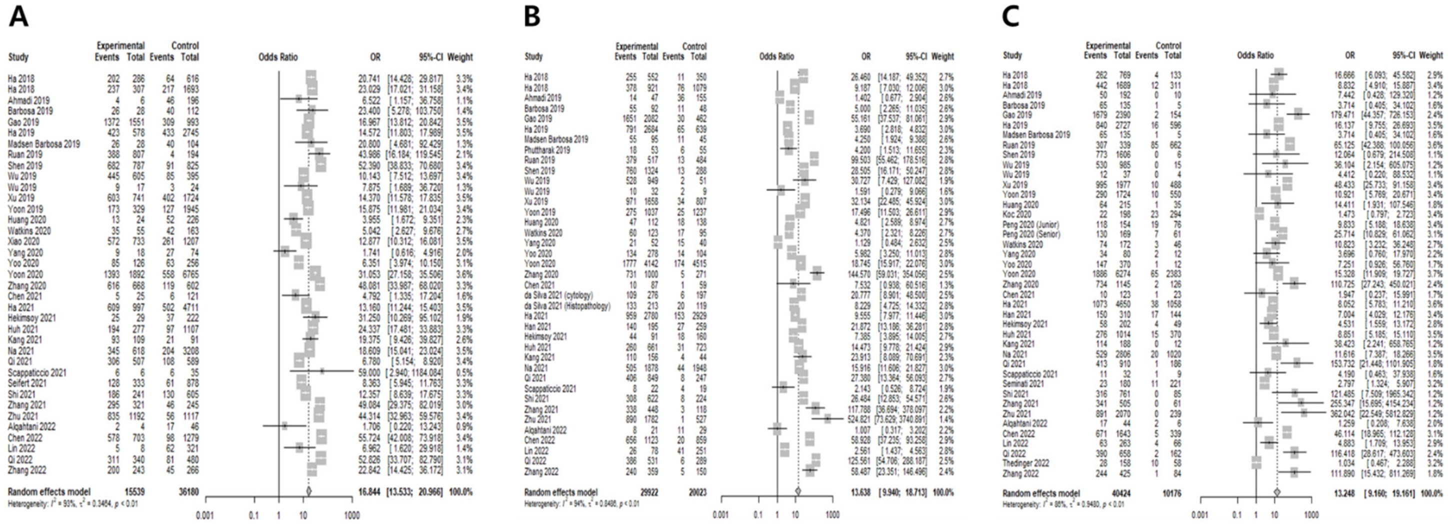

A. High

B. Intermediate

C. Low

Supplementary Figure S7. Summary receiver operating characteristic curve for ACR guidelines.

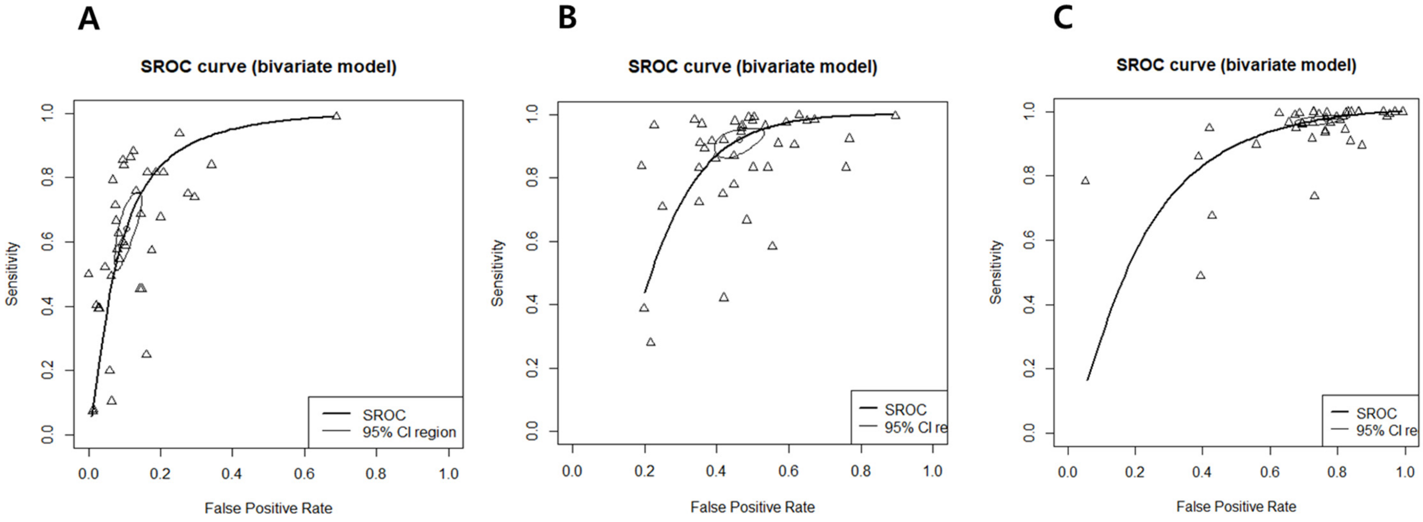

A. High

B. Intermediate

C. Low

Supplementary Figure S8. Forest plot of the diagnostic odds ratio for ATA

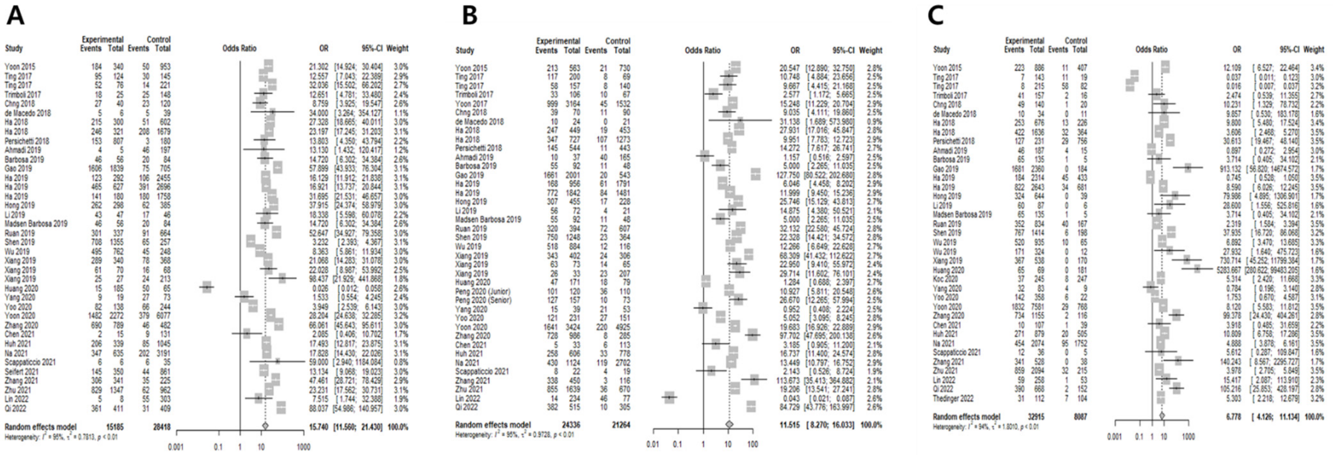

A. High
B. Intermediate
C. Low

Supplementary Figure S9. Summary receiver operating characteristic curve for ATA

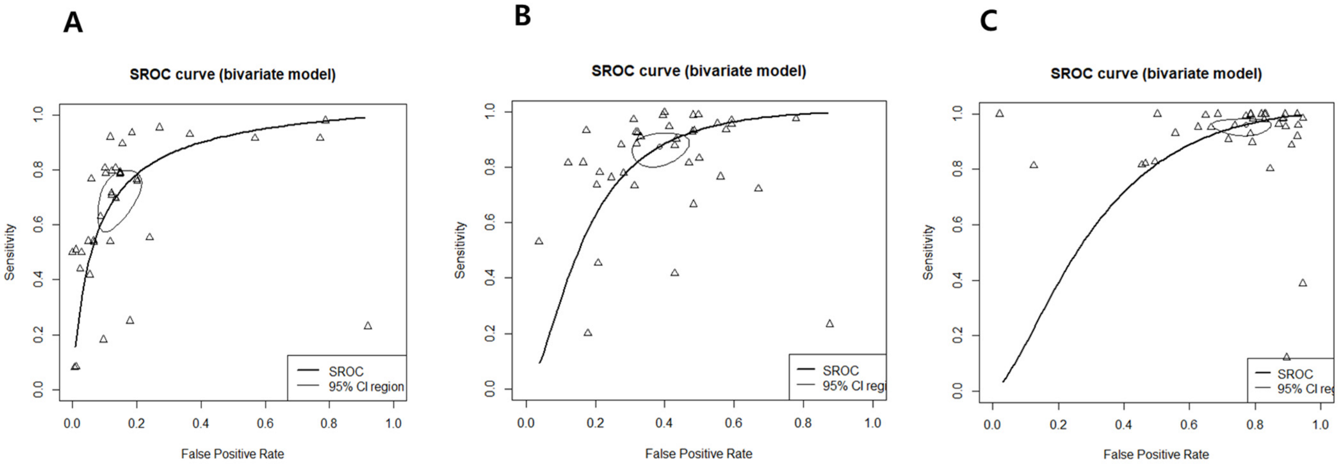

A. High
B. Intermediate
C. Low

Supplementary Figure S10. Forest plot of the diagnostic odds ratio for EU

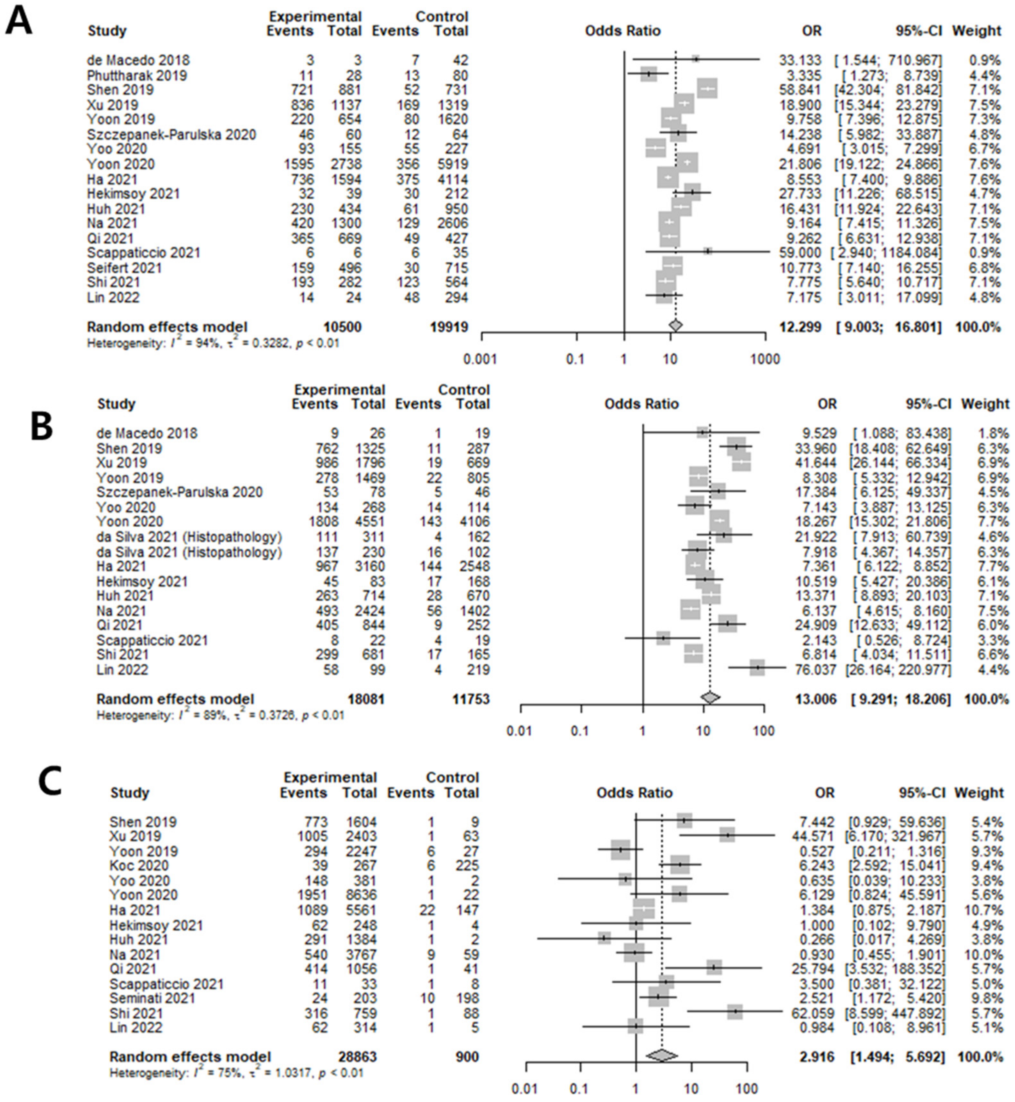

A. High

B. Intermediate

C. Low

Supplementary Figure S11. Summary receiver operating characteristic curve for EU

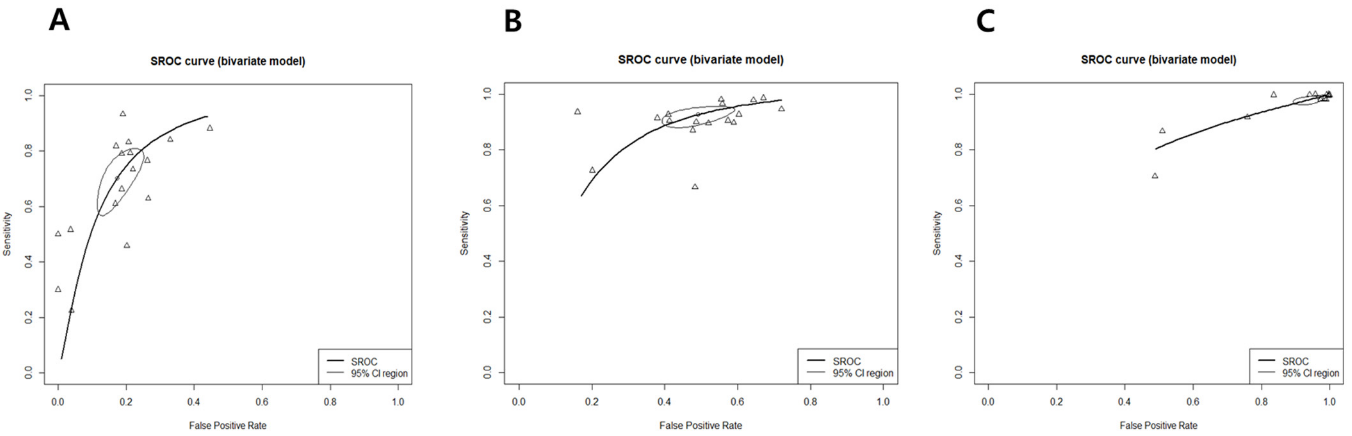

A. High
B. Intermediate
C. Low

Supplementary Figure S12. Forest plot of the diagnostic odds ratio for Kwak-TIRAD

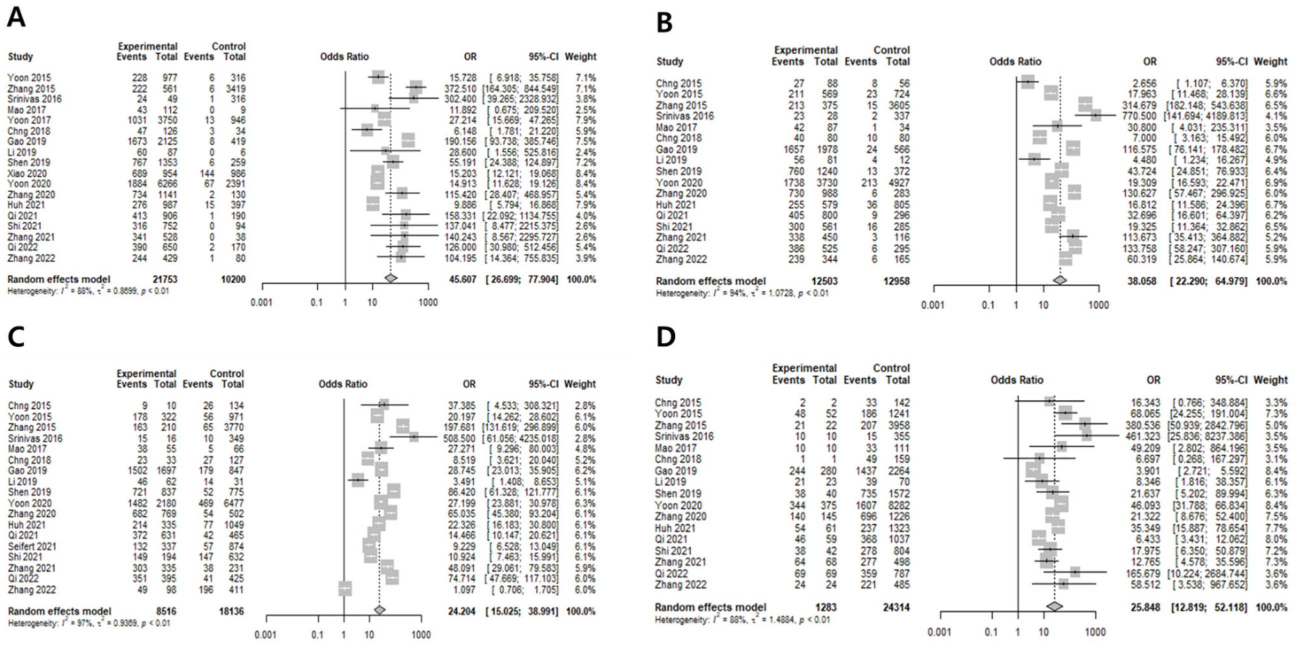

A. 4A

B. 4B

C. 4C

D. 5

Supplementary Figure S13. Summary receiver operating characteristic curve for Kwak-TIRAD

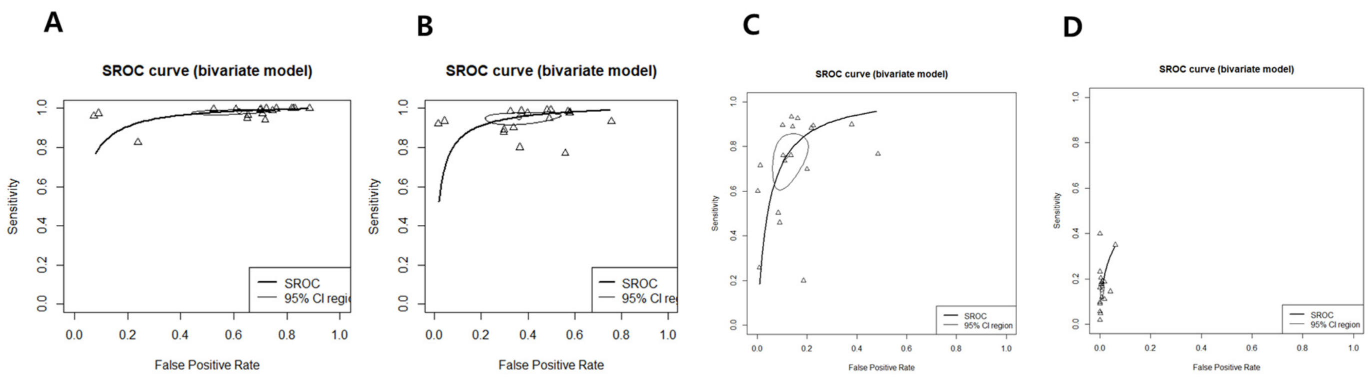

A. 4A

B. 4B

C. 4C

D. 5
